# Experience in Perioperative Management of Patients Undergoing Posterior Spine Fusion for Neuromuscular Scoliosis

**DOI:** 10.1155/2016/3053056

**Published:** 2016-12-12

**Authors:** Sébastien Pesenti, Benjamin Blondel, Emilie Peltier, Franck Launay, Stéphane Fuentes, Gérard Bollini, Elke Viehweger, Jean-Luc Jouve

**Affiliations:** ^1^Pediatric Orthopedics, Timone, Aix-Marseille University, 264 rue Saint Pierre, 13005 Marseille, France; ^2^Aix-Marseille University, CNRS, ISM, Inst Movement Sci, Marseille, France; ^3^Spine Unit, Timone, Aix-Marseille University, 264 rue Saint Pierre, 13005 Marseille, France

## Abstract

The objective of this investigation was to determine the outcome of spine fusion for neuromuscular (NM) scoliosis, using Unit Rod technique, with emphasis on complications related to preoperative general health. Between 1997 and 2007, 96 consecutive patients with neuromuscular scoliosis operated on with Unit Rod instrumentation were retrospectively reviewed. The inclusion criteria were diagnosis of NM scoliosis due to cerebral palsy (CP) and muscular dystrophy (DMD). Patient's preoperative general health, weight, and nutrition were collected. Different radiographic and clinical parameters were evaluated. There were 66 CP patients (59 nonwalking) and 30 DMD patients (24 nonwalking). Mean age at surgery was 16.5 years and 13.9 years, respectively. All radiographic measurements improved significantly. Wound infection rate was 16.7% (11% of reoperation rate in CP; 10% in DMD; 3 hardware removal cases). No pelvic fracture due to rod irritation was observed. Unit Rod technique provides good radiographic and clinical outcomes even if this surgery is associated with a high complication rate. It is a quick, simple, and reliable technique. Perioperative management strategy should decrease postoperative complications and increases outcome. A standardized preoperative patient evaluation and preparation including respiratory capacity and nutritional, digestive, and musculoskeletal status are mandatory prior to surgery.

## 1. Introduction

Patients with neuromuscular diseases frequently develop scoliosis that requires surgical correction [1–3]. Usually, spinal deformity is associated with great pelvic obliquity. Spine fusion in neuromuscular scoliosis aims to balance the trunk in frontal and sagittal plane, centre the head over the pelvis, and restore anatomical spine condition. Extension into the pelvis is meant to achieve global correction of both pelvic and spinal deformity [4–10].

A lot of instrumentation has been used in these specific deformities but the use of techniques such as the one described by Luque and Galveston remains the gold standard [10–13] with low complication rate related to the material, short operation time, and good functional results.

Patients with neuromuscular scoliosis undergoing posterior spinal fusion are at higher risk for postoperative complications due to underlying comorbidities [14–16] such as decreased pulmonary function, inadequate nutritional status, decreased mobility, and cognitive impairment. Complication rate associated with spinal surgery in neuromuscular scoliosis ranges from 17% to 74% [14, 16–24]. Few studies pointed out the relation between postoperative complications and preoperative nutritional, digestive, and respiratory preparation of patients.

The purpose of this study was to determine the outcome of spine fusion for neuromuscular scoliosis in a large consecutive series of patients with emphasis on complications related to preoperative general health.

## 2. Materials and Methods

It was a monocentric retrospective study. Between January 1997 and December 2007, 112 consecutive patients operated on for neuromuscular scoliosis in our department were reviewed. Inclusion criteria were (1) diagnosis of neuromuscular scoliosis consecutive to cerebral palsy (CP) or muscular dystrophy (DMD), (2) deformity correction by long posterior only spinal fusion according to the Unit Rod (UR) technique, and (3) minimum follow-up of 2 years. Excluded from this review were revision procedures, spinal fusions that did not extend to the pelvis, anterior or combined spinal fusion, other instrumentation, and other causes of neuromuscular scoliosis. In the final analysis, 96 patients met the inclusion criteria, 66 patients with CP and 30 with DMD. In CP group, 59 patients were nonwalking and 7 were ambulatory whereas, in DMD group, 6 patients were ambulatory. Among the included population, there were 62 males and 34 females.

**Figure 1:**
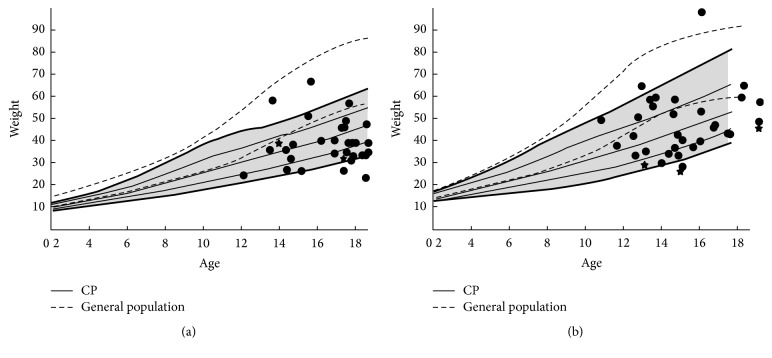
Growth curves comparing general population to cerebral palsy. 72% of the boys' weight (a) and 58% of the girls' weight (b) were below CP weight mean value, nonrelated to presence of gastrostomy (stars).

Medical records were used to assess preoperative weight and nutritional status using a growth curve comparing the weight of patients with CP to general population (Figure 1). If the patient's weight was not sufficient, a surgical or endoscopic gastrostomy was initially performed, usually 1 or 2 months before surgery. In addition to preoperative nutritional preparation, the gastrostomy was used in postoperative period for enteral nutrition in patients with alimentary difficulties. Other data were collected such as pre- and postoperative haemoglobin values (Hb), length of surgery, and intraoperative blood loss, need of red cell transfusion, length of intensive care unit, and hospital stay, in order to emphasise postoperative complications.

**Figure 2:**
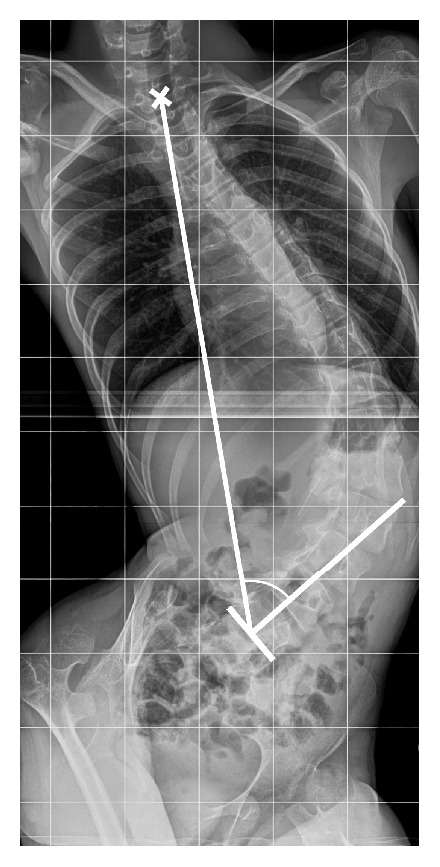
Measurement method of pelvic obliquity, defined as the angle between a perpendicular line to the sacroiliac line and the line joining the centre of T1 and the middle of the sacroiliac line.

Radiographic assessment was performed on anteroposterior pre- and postoperative radiographs, in sitting position. Evaluated parameters were Cobb angle, sacroiliac angle, and pelvic obliquity, defined as the angle between a perpendicular line to the sacroiliac line and the line joining the centre of T1 and the middle of the sacroiliac line (Figure 2).

Surgical complications were collected and divided into 3 categories: intraoperative complications, early postoperative complications, and late postoperative complications (hardware issues). Early postoperative complications were defined as events occurring during the first 21 days after surgery, late complications being events occurring after this period.

During surgery, blood pressure was monitored by a radial arterial catheter. A gastrojejunal tube was placed during surgical procedure and removed during 5 first postoperative days. An antiobioprophylaxy by Cefazolin was systematically performed at the beginning of surgery and repeated if the procedure lasted more than 6 hours.

**Figure 3:**
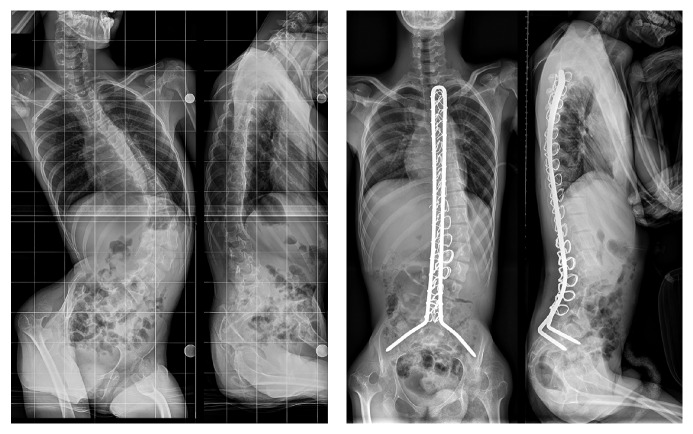
Pre- and postoperative radiography of long spinal fusion using Unit Rod technique in a patient with neuromuscular scoliosis due to cerebral palsy.

Surgical procedure consisted in long segment spinal posterior only fusion extended to the pelvis in all the cases (Figure 3). Patient was placed in prone position and a median approach was realized. Sublaminar wires were placed at each level. Four wires were used from L1 to L5 and two from the proximal end of the instrumentation to T12. In all cases, the fusion was performed from upper thoracic spine to pelvis, using 6.35 mm Unit Rod. Pelvic fixation was made as described by Galveston. For each patient, upper instrumented vertebra was located between T1 and T4 according to the initial spinal curve.

For statistical analysis, Student and Wilcoxon test were used. Data are presented as mean values and range. For continuous parameters, when the distribution was not normal, a Mann–Whitney nonparametric test was used. Results were considered statistically significant when *p* value was less than 0.05.

## 3. Results

Mean age at surgery in the whole population was 15.7 years. In CP group, mean age at surgery was 16.5 years (11 to 30 years) and 13.9 years (10.2 to 19.4 years) in DMD group.

Mean preoperative weight was 39.7 kg (19 to 84 kg). Thirty-four patients had preoperative denutrition or digestive concerns such as gastroesophageal reflux. Five of these patients had preoperative gastrostomy. Digestive and respiratory preoperative preparation was performed in three patients (noninvasive ventilation, physiotherapy, and colic preparation). One patient had colic preparation associated with physiotherapy, and two patients had noninvasive ventilation. Sixty patients had a specific postoperative alimentation, 56 of them had parenteral nutrition, 3 had continuous enteral nutrition, and one patient had both continue enteral and parenteral alimentation.

Preoperative radiographic assessment revealed a mean Cobb angle of 64°. Postoperatively, the mean Cobb angle was 25°. The difference was statistically significant (64° versus 25°, *p* < 0.01). The mean Cobb angle correction rate was 61.5%. Pelvic obliquity also decreased significantly, from 17.5° preoperatively to 5° postoperatively (*p* < 0.01). The mean pelvic obliquity correction rate was 73.5%. With regard to sagittal alignment, thoracic kyphosis and lumbar lordosis were not significantly modified between pre- and postoperative evaluation. However, DMD patients had significantly lower sagittal curvatures than CP patients (Table 1). Furthermore, 20 patients in the CP group required prior hip surgery (hip repositioning procedures) and none in the DMD group.

Mean surgery time was 277.8 minutes. Mean intensive care unit stay was 2 days. Preoperatively, mean Hb was 13 g/dL and 9.5 g/dl postoperatively, without significant difference between CP and DMD groups (*p* = 0.09). Red cell transfusion was required in 33 patients (35.1%). On average, first postoperative oral feeding was at day 6 on the whole series, significantly (*p* = 0.02) quicker (day 4) in the DMD group than in the CP group (day 7). Average length of hospital stay was 15.7 days.

Intraoperatively, 3 complications occurred (3.2%), only in cerebral palsy group. There were 2 cardiopulmonary arrests and 1 dural tear.

Early postoperative complications occurred in 40 of the 96 patients (42.6%) and are summarized in Table 1. Two patients died postoperatively (2,1%): one patient died from unexplained heart failure 24 hours after the intervention and one patient died 4 days after surgery from a probable gastric perforation. Thirty-one infectious complications were diagnosed (32.3%), including 16 wound infections (16.7%). Among these infections, 7 in the CP group and 3 in the DMD group were deep infection below the spinal fascia and required surgical debridement. All wound infections were caused by gastrointestinal germs and were associated with a lower body weight and poor nutritional status. Six patients were treated by antibiotherapy alone and 10 patients underwent debridement surgery associated with antibiotherapy. In any case hardware removal was necessary during the first 3 months. The 15 other infections were 7 cystitis, 4 pneumonia, and 4 septicemia cases. Other general complications were 2 atelectasis, 3 respiratory distress, and 3 reactive ileus cases.

Seventeen patients (18.1%) had late postoperative complications (Table 1). Fourteen late complications were due to hardware: 11 patients had a windshield wiper effect in the ilium and 3 patients had a rod fracture. In 4 cases (4.2%), hardware removal was necessary: for 3 patients with recurrent wound infections with a fused spine (without further modification of spinal curves) and one patient with a rod fracture that required a revision procedure with pedicular screws.

## 4. Discussion

Neuromuscular scoliosis is complex and still challenging with regard to the type of spinal deformity and patients' general medical condition [25]. This kind of surgery is often associated with high mechanical complication rate as hardware fracture, tearing of sacral fixation, loss of lumbar lordosis, and a significant rate of pseudarthrosis [26, 27]. Thus, interest of Unit Rod is to provide a segmental fixation, allowing a good distribution of constraints all along the spine. The Unit Rod is an extremely resistant autostable instrumentation, avoiding postoperative restraint. It is quick and simple to use, although it is technically more difficult in patients with hyperlordosis. It is considerably less expensive than most other systems. The Unit Rod can achieve good deformity correction with a low loss of correction, as well as a low prevalence of associated complications and reoperation rate [26–28]. In our series, reoperation rate related to implant failure was only at 4.2%.

Correction achieved by Unit Rod was satisfactory in our population, with Cobb angle correction rate of 61.5% and pelvic obliquity correction rate of 73.5%. These results are comparable to those found in literature, with Cobb angle correction rates ranging from 54 to 82% and pelvic obliquity correction rate ranging from 42 to 86.8%, using Luque Galveston or Unit Rod instrumentation [25, 29–32].

As an alternative to LG instrumentation and associated techniques, some authors have described the use of Cotrel-Dubousset (CD) instrumentation in neuromuscular scoliosis [9, 33, 34]. Comparison of LG instrumentations in neuromuscular scoliosis does not reveal differences in terms of radiological outcome, complications, and patient satisfaction in the literature [18]. The mean operating time in our series was 277.8 minutes, which is comparable to those reported by authors using CD instrumentation [9, 34].

Recently, multilevel instrumentation with all-screw construct has been described for neuromuscular scoliosis [35, 36]. Resistance to pull-out constraints is theoretically improved with this kind of instrumentation but specific mechanical complications have been described, such as surrounding osteolysis around screws [37, 38]. Another theoretical advantage of Unit Rod technique can be related to the low price of the implant. As an example, in France a Unit Rod costs between 200 and 500 euros while each pedicular screw costs around 150 euros.

Complication rate after surgical correction of neuromuscular scoliosis is variable according to different authors but remains high [14, 16–21, 24–27, 29]. In these different studies, complication rate ranges from 17 to 74%, Benson et al. [21] reporting the highest rate with 17 complications in 24 patients, predominantly infectious and respiratory problems. Our results are consistent with an overall early complication rate of 50%. Curve magnitude and nonambulatory status have been described as risk factors of major postoperative complications [17].

Among complications, prevalence of wound infections ranges from 8.7% to 20% [17, 39–41]. Degree of patients' cognitive impairment, denutrition, respiratory problems, and intraoperative bleeding are associated with an increased infectious rate [40, 42–44]. In our series, wound infection was diagnosed in 16.7% of the population. Six patients were treated using antibiotics alone, and the other 10 required a reoperation associated with antibiotherapy. In first intention, wound infections treatment after spinal surgery must be conservative, and hardware removal must be considered only if infection persistence is diagnosed after an appropriate treatment [39].

Respiratory complications are a major concern in these patients, occurring in 23.5 to 57% of cases [21, 29, 45]. The analysis of our series found respiratory complications in only 15.6% of the population. This observation may be the consequence of the respiratory preparation patients underwent before surgery (noninvasive ventilation and physiotherapy). It has been previously proved that patients' preoperative general status was correlated to complication rate [46]. Thus, we believe that respiratory preparation is of major importance in these patients' management [47–50].

Digestive complications remain relatively rare in the literature [21, 29, 51]. However, this kind of complications may be serious. In our series, one patient died from a probable gastric perforation. In the same way, Master et al. [17] reported major gastrointestinal complications with 2 cast syndrome cases and 1 case of concomitant gallbladder hydrops and pancreatitis. Risk factors for digestive complications are hypotensive anaesthesia [31], intraoperative position, and denutrition, especially in cast syndrome occurrence [42, 52].

Most of late complications were due to hardware failures including 13 pseudarthrosis cases that led to 3 rods breakage and 3 proximal junctional kyphosis cases. Most studies report one or two cases of rod fracture [26, 30, 32]. Nectoux et al. [29] did not report reintervention due to major mechanical complications although 10 asymptomatic cases of windshield wiper effect occurred in the long term.

Compared with idiopathic scoliosis, neuromuscular scoliosis patients requiring spine surgery have a higher risk of adverse perioperative complications because of underlying comorbidities [14, 51]. Comorbidities commonly associated with neuromuscular scoliosis are decreased pulmonary function, inadequate nutritional status, decreased mobility, and communication and cognitive impairment.

## 5. Conclusion

Unit Rod technique is quick, simple, reliable, and probably less expensive than other techniques. This strategy offers satisfactory deformity correction. During the last decade, improvement of patients' care has permitted underlining the crucial importance of managing spinal deformities during adolescence or early adulthood. Nevertheless there are still numerous patients in poor general condition presented for spine surgery.

Spinal fusion in neuromuscular scoliosis is exposed to a high complication rate, correlated to preoperative general health and respiratory status. A standardized preoperative patient evaluation and preparation including respiratory rehabilitation and nutritional care are mandatory prior to surgery. Perioperative management strategy may decrease postoperative complications and increases outcomes.

Nonwalking neuromuscular patients are supposed to need spine surgery. Best long-term general follow-up should be performed in these patients. Orthopedic surgeons have to be associated with global management program of these patients.

## Figures and Tables

**Table 1:** Statistical comparison between CP and DMD patients.

	CP		DMD		*p*
	*N*	%	*N*	%	
Number of patients	66	—	30	—	
Age at surgery	16.5	—	14,2	—	*0,001*
Ambulatory	7	11%	6	20%	*NS*

Preoperative Cobb angle	69,7	—	39,1	—	*<0,001*
Postoperative Cobb angle	25	—	13	—	*<0,001*
*Correction rate*	*59,6%*		*79,4%*		*0,001*
Preoperative pelvic obliquity	17,7	—	12,8	—	*NS*
Postoperative pelvic obliquity	5,8	—	2,2	—	*0,006*
*Correction rate*	*64,5%*		*87,7%*		*0,003*
Preoperative TK	35,3	—	6,2	—	*<0,001*
Postoperative TK	30,2	—	6,8	—	*<0,001*
Preoperative LL	40	—	15	—	*<0,001*
Postoperative LL	40,9	—	22,3	—	*<0,001*

Early complications	39	59%	9	30%	*0,02*
*Wound infection*	13	20%	3	10%	*NS*
*Deep infection that required surgical debridement*	*7*	*11%*	*3*	*10%*	*NS*
*Other infections*	10	15%	5	17%	*NS*
*Gastrointestinal issues*	6	9%	1	3%	*NS*
*Other*	2	3%	0	0%	*NS*
*Mortality*	2	3%	0	0%	*NS*

Hardware issues	9	14%	7	23%	*NS*
*Pseudarthrosis*	7	11%	6	20%	*NS*
*PJK*	2	3%	1	3%	*NS*
*In which return to OR*	*3*	*33%*	*1*	*14%*	*NS*
